# Ultrafast Dynamics and Rearrangement of the EUV Photoacid Generator Phenyl Triflate

**DOI:** 10.1021/acs.jpclett.4c03621

**Published:** 2025-03-27

**Authors:** Sung Kwon, Jacob Stamm, Marcos Dantus

**Affiliations:** † Department of Chemistry, Michigan State University, East Lansing, Michigan 48824, United States; ‡ Department of Physics and Astronomy, Michigan State University, East Lansing, Michigan 48824, United States; ¶ Department of Electric and Computer Engineering, Michigan State University, East Lansing, Michigan 48824, United States

## Abstract

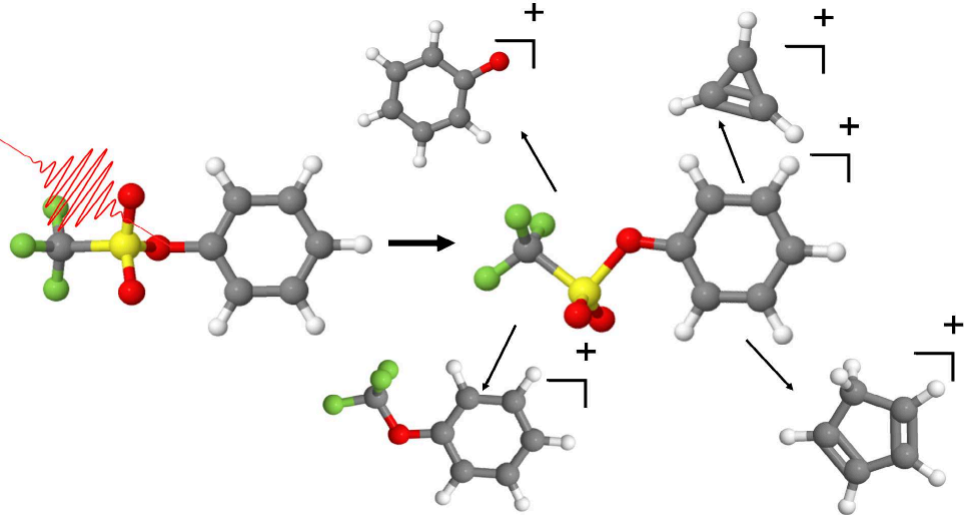

Extreme UV photolithography is a high-energy process crucial for modern computer chip fabrication, where photoacid generators (PAGs) enhance radiation exposure by producing reactive chemical species. This study investigates the ultrafast dissociative dynamics of phenyl triflate, a widely used PAG, under high-energy ionizing conditions. The dissociative ionization of the phenyl triflate cation, which includes a molecular rearrangement that releases SO_2_ and forms the phenyl trifluoromethyl ether cation, is measured over the first 5 ps. These dynamics reveal vibrational coherence corresponding to a torsional mode of the cation, involving the twisting of the phenyl group into the O–S–C plane. Electronic structure calculations for the radical cation are in good agreement with the experimentally observed vibrational coherence. These findings provide valuable insights into the behavior of phenyl triflate under ionizing conditions similar to its industrial usage.

The semiconductor industry has adopted extreme ultraviolet (EUV) photolithography, which currently uses 13.5 nm light to create intricate patterns on photoresist materials applied to semiconductor wafers. This technology has been crucial for maintaining Moore’s law, the trend of doubling transistor density on chips approximately every two years. As EUV interacts with the photoresist, it releases photoelectrons that ionize the film or underlayer, generating additional secondary electrons.^1−4^ The shift to shorter wavelengths in microchip technologies has garnered interest in the chemistry of photoacid generators (PAGs), such as phenyl triflate (PTF), for use in chemically amplified photoresists at these wavelengths. Upon ionization by the secondary electrons, PAGs release acidic groups that drive deprotection and cross-linking, altering the solubility of the exposed regions. These regions are then either washed away (in positive tone photoresists) or left intact while the unexposed regions are washed away (in negative tone photoresists), leaving behind patterns that can be further processed through doping, etching, or metal filling to construct the chip’s intricate three-dimensional structure. However, the high photon energy of EUV light (92 eV) introduces significant challenges in understanding and predicting the far-from-equilibrium chemistry involved.^4−6^ Studying photoresists in their native condensed phase is challenging,^5−7^ but gas-phase mass spectrometry has proven effective for determining EUV photoresist fragmentation in commercially available halogenated methylphenol analogs of monomer photoresists,^8^ organic EUV-resist monomers,^9^*tert*-butyl methyl methacrylate photoresist monomers,^10^ potential photoresist 2-(trifluoromethyl)acrylic acid,^11^ and PTF, a PAG utilized in modern computer chip fabrication.^6^ Laffert et al. investigated the products following the dissociative photoionization of PTF in the gas phase at 92 eV using synchrotron radiation.^6^ They proposed that no fragmentation channel led to the formation of triflic acid, the compound expected to control photoresist solubility. They observed a sequential loss of SO_2_, CF_3_, CO, and C_2_H_2_, and their findings were supported by electronic structure calculations.

Our group has been exploring the ultrafast chemical processes that occur following the interaction of molecules with secondary electrons.^12,13^ Strong-field ionization (SFI), which generates ions with a wide range of internal energies, effectively simulates the chemical activation seen in EUV photolithography, where secondary electrons with a broad range of energies play a key role.^1−3^ Here, the ultrafast dissociative dynamics of ionized PTF are examined with femtosecond time resolution. Our findings offer new insights into the mechanisms at play within photoresists under high-energy conditions, with a particular emphasis on the ultrafast chemistry occurring immediately after ionization, before collisions with nearby molecules become important. The experimental SFI mass spectrum of PTF at an intensity of 1.1 × 10^14^ W/cm^2^ is shown in Figure 1.

**Figure 1:**
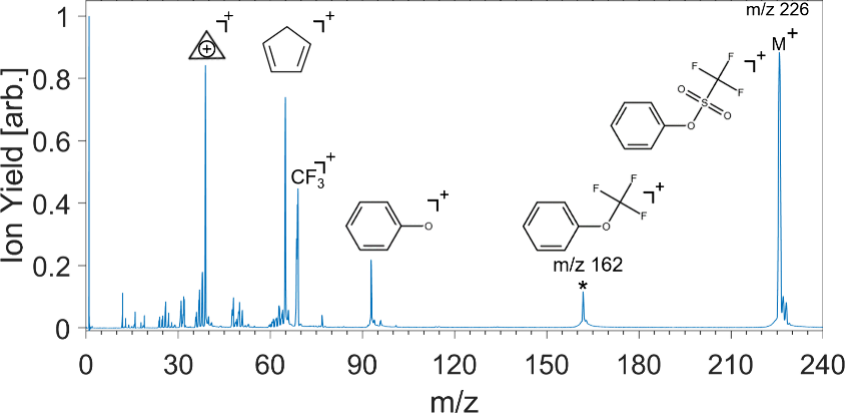
Experimental SFI mass spectrum of phenyl triflate. The structure of the major fragments are shown. The rearrangement product is indicated with an asterisk.

Notably, smaller ring structures such as cyclopropenium (*m*/*z* 39) and cyclopentadiene (*m*/*z* 65) are similar to those formed during the dissociative ionization of toluene and nitrotoluene.^14^ The rearrangement product at *m*/*z* 162 is generated from the molecular ion through the loss of SO_2_, with the CF_3_ group bonding to the oxygen.^6^ This fragment ion is observed in the mass spectrum even at low intensities. Additionally, the absence of a fragment ion at *m*/*z* 157, which would correspond to the direct loss of CF_3_, and *m*/*z* 133, which would correspond to the loss of the triflate ion, further support the prevalence of the intramolecular rearrangement. Differences between the spectra obtained here and those from 92 eV single-photon excitation,^6^ including the greater abundance of the molecular ion and cyclopropenium, stem from the broad range of intramolecular energies produced by SFI. Recall that in EUV photolithography the majority of chemical transformations are induced by secondary electrons^1−3^ and that the electron cross section peaks at 70 eV. To mimic 70 eV electron–ionization mass spectra, it has been found that one needs 20 eV photons.^15^ We propose that SFI is best suited for mimicking the broad range of ionization energies in EUV photolithography. Intense femtosecond near-IR pulses meet this requirement and, when combined with disruptive probing,^16^ can provide valuable insights into the fragmentation dynamics of PAGs like PTF. In this pump–probe technique, a strong pump pulse ionizes the molecule, while a weaker, nonionizing probe pulse disrupts product formation. The probe pulse is scanned with a variable time delay relative to the pump. By measuring the ion yield of all fragments, the resulting dynamics of each fragment ion can be fitted to the following equation:^16^
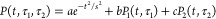
1where
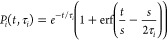
2

For the above equation, *a*, *b*, and *c* are the amplitude factors, τ_*i*_ is a time constant defining a decay (τ_*decay*_) or rise (τ_*rise*_) of the signal, *t* is a specific pump–probe delay, and *s* is a parameter related to the full width at half-maximum (fwhm) of the pulse duration by
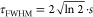
3The time-dependent yield of all major fragment ions shows a rise or decay that was fit to eq 1 by three exponential components described by eq 2, together with fast-dephasing vibrational coherence. The molecular ion yield, shown in Figure 2a, is depleted by the probe pulse as it induces dissociation into the multiple fragments. The *m*/*z* 162 ion yield resulting from the loss of SO_2_ (Figure 2b) shows an early depletion that partially recovers within a picosecond. The cyclopentadienyl cation, Figure 2c, as well as all other major fragments, shows coherent dynamics that appear to have oscillations matching those of the molecular ion, indicating the probe pulse induces transitions leading to the various products. The time-resolved (−0.5 to 9 ps) data for m/z 39, 65, 69, 93, 162, and 226 is shown in Figure S1. The time constants of the fits shown in Figure 2 are summarized in Supporting Information Table S1. The fastest dynamics correspond to a 160 fs rise in *m*/*z* 162, that we hypothesize is related to the phenyl group rotation into the O–S–C plane. The remaining time constants relate to the 0.5–5 ps dissociation dynamics of this large molecule. A more detailed description of the dynamics will require higher level calculations, and ab initio molecular dynamics simulations. We consider this molecule to be an ideal candidate to be examined by ultrafast X-ray or electron diffraction methods, which are the only experimental tools capable of capturing the motion of the entire molecular structure. Efforts along these lines are already being planned.

**Figure 2:**
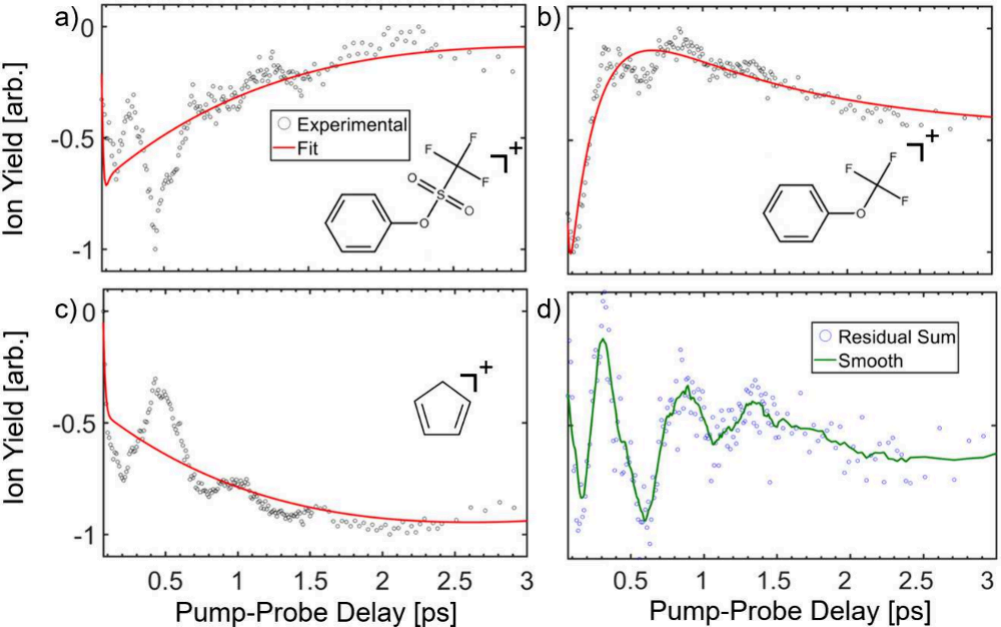
Time-resolved ion yields and fits to eq 1 for *m*/*z* 226 (a), *m*/*z* 162 (b), and *m*/*z* 65 (c) as well as the sum of the residuals for major fragments *m*/*z* 39, 65, 69, 93, and 162 (d). The data were normalized so the baseline ion yield is zero and minimum ion yield is −1. The intensities of the pump and probe pulses were 1.1 × 10^14^ W/cm^2^ and 3.0 × 10^13^ W/cm^2^, respectively. The summed residuals were fit to a decaying exponential to eliminate asymmetry in the oscillations. A ten-point moving average was plotted to guide the eye.

The oscillations observed were isolated by subtracting the fit from eq 1 from the experimental data to obtain residuals. We summed the residuals of the major fragments corresponding to *m*/*z* 39, 65, 69, 93, and 162, shown in Figure 2d, to improve the signal-to-noise ratio. The individual residuals for all major fragments are shown in Figure S2. The fast Fourier transform (FFT) and the maximum entropy method (MEM)^17^ were used to extract the oscillation frequencies. The MEM was chosen for its superior resolution in analyzing few-cycle oscillations compared to FFT. The peak at 64 cm^–1^ observed in the FFT is also present in the MEM results (see Figure 3). The coherence mapping method,^18^ which compares the observed vibrational frequencies across product ions to elucidate the intermediate(s) they originate from, was used to analyze the main products. We observe that most ions are modulated by two frequencies corresponding to ∼ 33 and 64 cm^–1^. Phase analysis was also carried out (see Figure S3) and results are summarized in Table 1. The 64 cm^–1^ oscillations, with a period of 521 fs, show a phase shift that increases as fragment size decreases (see Table 1), ranging from 1.5 π to 0 π and corresponding to a delay of approximately 390 fs (see Figure S2). Production of the smaller fragment ions requires overcoming barriers of increasing height,^6^ and thus requires relaxation from higher excited states with different relaxation.

**Table 1:** Phase Analysis Results for *m*/*z* 226, 162, 93, 69, 65, and 39^a^

*m*/*z*	Frequency 1 (cm^–1^) ± 4	Phase 1 (π) ± 0.1 (33 cm^–1^)	Frequency 2 (cm^–1^) ± 4	Phase 2 (π) ± 0.1 (64 cm^–1^)
226	33	2.0	64	1.5
162	25	1.9	64	1.0
93	–	–	63	0.5
69	41	1.0	65	0.4
65	28	1.0	56	0.4
39	41	1.0	72	0.0

a The Fourier transform power and phase as a function of frequency for these ions are given in Figure S3.

**Figure 3:**
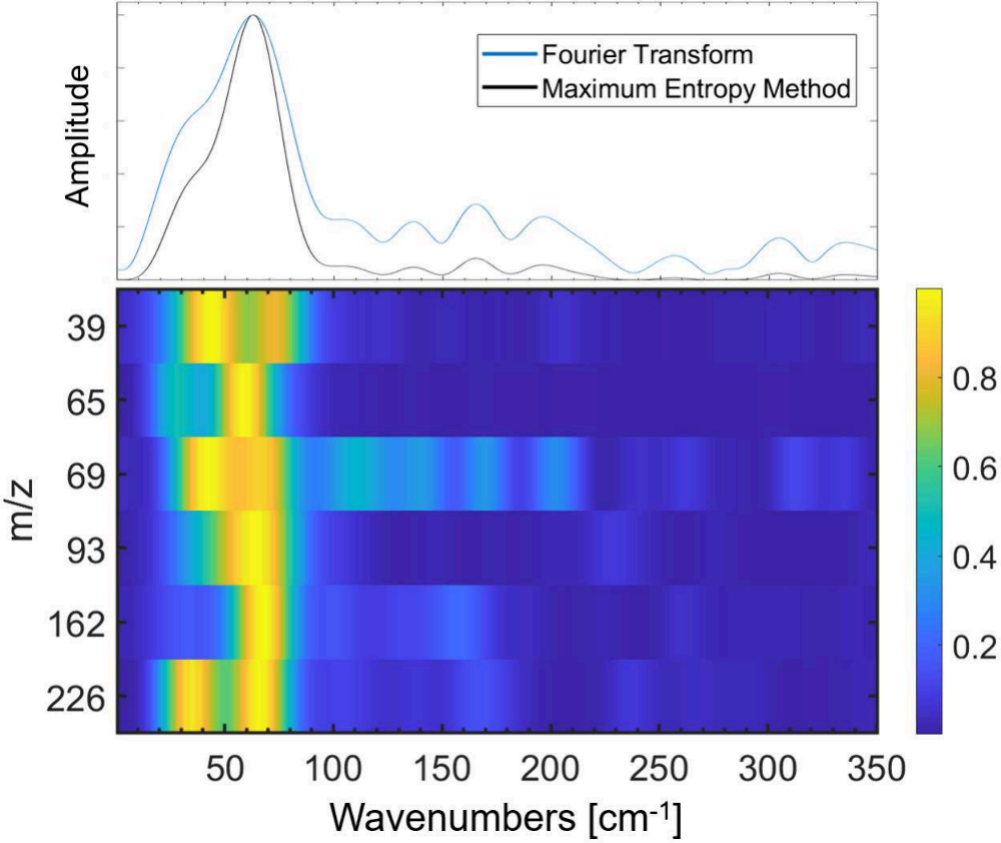
Top panel shows the experimental summed FFT and MEM spectra of the major fragments and molecular ion (*m*/*z* 39, 65, 69, 162, and 226) with the coherence map. The bottom panel shows the coherence map of the major fragments. The peak at 64 cm^–1^ matches for both the FFT and MEM and is prominent for the major fragments in the coherence map.

Quantum chemical calculations of critical geometries of the neutral and singly charged PTF molecule were performed using the ωB97X-D functional with the 6-311+G** basis set to identify vibrational modes that correspond to the experimentally measured coherent motion and the results are shown in Figure 4. The neutral ground state of PTF has its phenyl ring rotated 90° relative to the O–S–C plane. This is expected to be the dominant conformer of neutral PTF based on the relative energies of different phenyl dihedral angles relative to *kT* at room temperature (see Figure S4). Upon ionization, the phenyl ring rotates to align with the O–S–C plane. This rotational motion causes a significant geometric distortion, releasing 0.75 eV of excess energy to the molecule.

**Figure 4:**
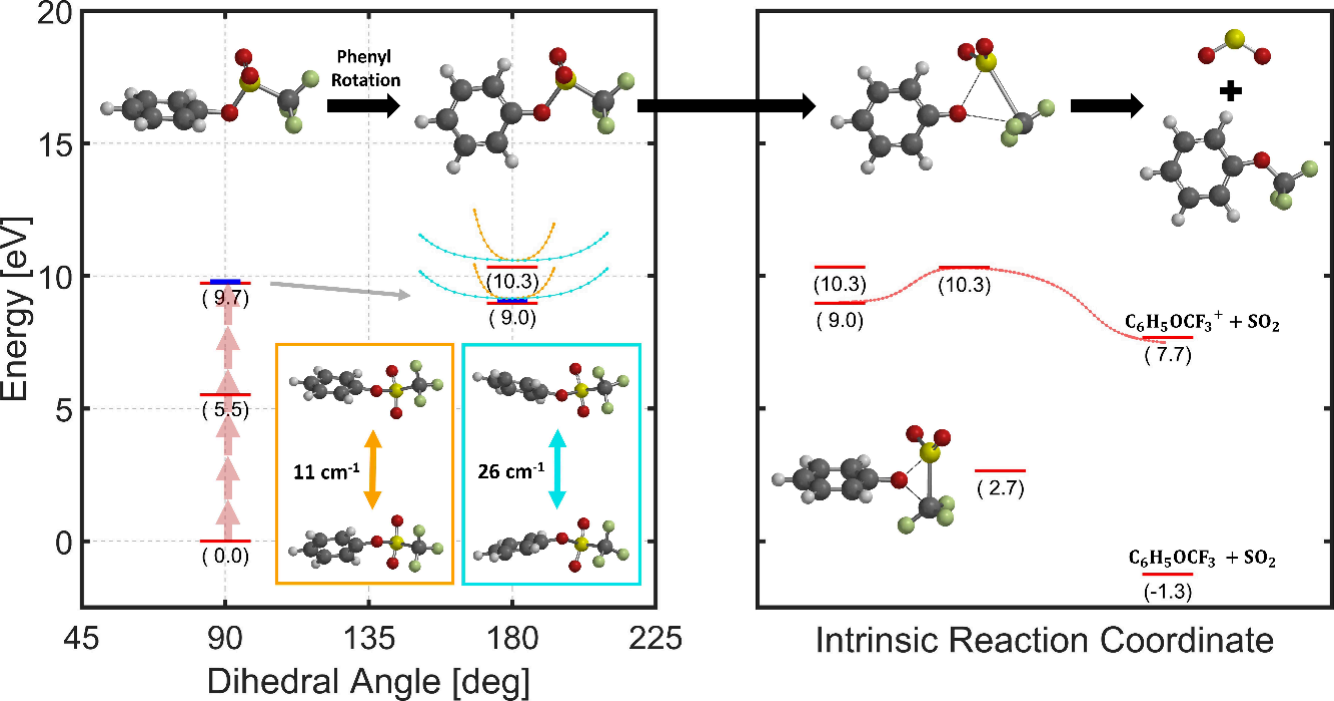
Calculated potential energy surface of phenyl triflate upon ionization. The pathway leading to SO_2_ loss is broken into two steps: rotation of the phenyl group (first panel) and the intrinsic reaction coordinate associated with the SO_2_ loss transition state geometry (second panel). The ground and excited state energies of the first two ground vibrational modes of the PTF cation are shown as cyan and orange lines. The energy of states along the intrinsic reaction coordinate for SO_2_ loss are shown as a red line. All energies were calculated using the ωB97X-D/6-311+G** level of theory and expressed relative to the ground state of the neutral molecule. Blue lines correspond to identical calculations but performed with the MP2/cc-PVDZ level of theory.

The 90° dihedral rotation upon ionization activates vibrational modes of the cation involving a twisting motion of the phenyl ring. Such motion modulates the transition probability induced by the probe at twice the oscillation frequency because the wavepacket visits the resonant geometry twice during the vibrational period. We identify the resonant geometry as the planar configuration of the cation, when the probe can induce a one-photon transition to the first excited state of the PTF cation. When the phenyl ring is twisted, causing the probe to be out of resonance, excitation becomes less likely, and the molecule does not have enough energy to eliminate SO_2_, so it preferentially remains as a molecular ion. However, when the phenyl ring lies in the plane of the O–S–C atoms, the fragmentation pathway to eliminate SO_2_ can proceed, as the barrier is overcome by the probe energy. The observed phase shift in the 64 cm^–1^ oscillations of the different fragment ions (Figures S2 and S3, and Table 1), which decreases as fragment size diminishes, is hypothesized to be related to the ∼390 fs required for relaxation from the higher excited states induced by the pump to the cation’s ground state. The formation of smaller fragment ions requires overcoming progressively higher barriers,^6^ suggesting they are produced as higher cationic excited states relax over longer times.

In the PTF cation, the two lowest frequency vibrational modes, with frequencies 11 and 26 cm^–1^, involve the phenyl torsional motion and are expected to be responsible for the oscillations observed in the pump–probe transients. Given that the wavepacket visits the resonant geometry twice during the vibrational period we double the calculated frequencies to obtain 22 and 52 cm^–1^, both of these being in reasonable agreement with the experimental values 33 and 64 cm^–1^. These vibrational modes modulate the resonance of the probe pulse with an excited state, identified to be the first excited state of the cation which is close to a 1-photon resonance by the probe when near the cation minimum, but out of resonance when the phenyl ring is rotated away from this position (see Figure S5). Excitation of the PTF cation by the probe provides enough energy (especially when combined with the 0.75 eV excess energy) to surpass the transition state barrier for SO_2_ ejection. Therefore, the modulations induced by the vibrational modes of the cation also modulate SO_2_ elimination and C_6_H_5_OCF_3_^+^ formation.

A previous study^6^ proposed a mechanism in which SO_2_, CF_3_,CO, and C_2_H_2_ are cleaved sequentially from the molecular ion resulting in the remaining fragmentation products *m*/*z* 162, 93, 65, 39. The similar modulation frequency observed in the yield of these products (Figures 2 and 3) is consistent with this mechanism because it indicates a common transition state barrier acting as a bottleneck for the subsequent fragmentation.^18^

Finally, it is worth discussing the nature of the identified transition state (Figure 4). This geometry places the CF_3_ group 2.79 Å from the oxygen, which is unexpectedly farther than the ground-state equilibrium cation geometry (2.63 Å). The molecular rearrangement that results in C_6_H_5_OCF_3_^+^ requires cleavage of two bonds and formation of one bond. As we explore the calculated transition state geometry we notice SO_2_ moves away, while the CF_3_ moiety adopts a quasi-planar geometry that is reminiscent of the equilibrium structure of CF_3_^+^. We explain this as the CF_3_ group donates a bonding electron to the SO_2_ and attacks the lone pair on the C_6_H_5_O group. This process likely benefits from the O–S–C bending mode, which we calculate near 160 cm^–1^. We find evidence of this motion in the coherence map shown in Figure 3 for *m*/*z* 162. The rearrangement likely occurs in an asynchronous concerted process, as cleavage of the O–S bond would lead to the dissociation of SO_2_CF_3_^+^, a fragment that is not observed in the mass spectrum. This is in contrast to the calculated transition state for the same SO_2_ loss identified in the neutral molecule, where the geometry shows a smaller (2.08 Å) O–C distance with a nonplanar CF_3_ geometry. This raises the possibility of a concerted mechanism for the intramolecular rearrangement in the neutral molecule (given sufficient energy). Such a difference between neutral and cation mechanisms could be related to the difference in geometric structure of the C_6_H_5_OCF_3_ cation, where the CF_3_ is in the plane of the phenyl group, compared to the neutral where it is out of the plane. Further experiments, perhaps including ultrafast electron/X-ray diffraction as well as higher level computational work, are needed to ascertain the concertedness of the SO_2_ loss mechanisms in both the neutral and cationic PTF.

In conclusion, this study provides valuable insights into the ultrafast dynamics of the dissociative ionization of the PTF cation under high-energy conditions similar to those in EUV photolithography. Our findings show that the dissociative ionization of phenyl triflate leads to molecular rearrangement and loss of the SO_2_ group. Various fragment ions exhibit vibrational coherence associated with a torsional mode that brings the phenyl ring into the O–S–C plane. Electronic structure calculations of the radical cation confirm the frequency of the torsional mode, which aligns well with the experimentally observed vibrational frequencies. These results enhance our understanding of the behavior of phenyl triflate under high-energy conditions, shedding light on its role in photolithography and chip fabrication processes. In the condensed-phase environment of a chemically amplified photoresist containing PTF, the highly reactive nascent fragments generated by high-energy ionization from EUV photons and secondary electrons modify the photoresist’s solubility.

## Methods

The experimental methodology used to track all reaction pathways after SFI has been described elsewhere.^16^ Briefly, a Ti:sapphire laser with a central wavelength of 795 nm was utilized as the ionization source. Phenyl triflate was purchased from Sigma-Aldrich (98% purity) and used without further purification. The pulses were compressed using the Multiphoton Intrapulse Interference Phase Scan (MIIPS) method,^19^ which measured and compressed the pulses to 40 fs by the MIIPS-HD pulse shaper. The laser pulses were focused into a Wiley–McLaren time-of-flight (TOF) mass spectrometer via a 200 mm lens and polarized parallel with respect to the TOF. Each laser pulse was split into a strong pump pulse, which ionizes the molecule, and a weak probe pulse, which disrupts the formation of products. The intensities were calibrated by measuring the ratio of Ar^2+^ to Ar^+^ ions.^20^ The PTF vapor was introduced into the TOF chamber as an effusive beam through a needle valve. The static pressure inside the chamber during data acquisition was maintained at 1 × 10^–5^ Torr. The baseline vacuum pressure as 9 × 10^–8^ Torr and the pressure returned to baseline within seconds when the needle valve was closed. The ion signals were digitized using an oscilloscope (LeCroy WaveRunner 610Zi, 1 GHz). Lastly, the TOF was configured to measure cations, thus only positive ions are shown in the mass spectra.

All theoretical calculations were performed with Spartan ’24 (V1.2.0). Ground state neutral and cationic structures were calculated via the ωB97X-D method using the 6-311+G** basis set. Transition state structures were determined via a mixture of constrained optimizations, comparison to similar molecular transition states, and finally optimization using the above level of theory, verifying the structure contains a single imaginary frequency component.

## Supporting Information Available

Ion yield vs pump–probe time delay for major fragment ions, residuals after fitting showing coherent oscillations, table with fit parameters, FFT magnitude and phase for major fragment residuals, energy of neutral and cationic PTF vs phenyl group rotation, and energy difference between the ground and first excited states of the PTF cation as its geometry changes according to the three lowest vibrational modes (PDF)
Transparent Peer Review report available (PDF)
