# Supplementary material for: Ultrafast Dynamics and Rearrangement of the EUV Photoacid Generator Phenyl Triflate
Source: J Phys Chem Lett. 2025 Mar 27;16(13):3397–401. doi: 10.1021/acs.jpclett.4c03621 (PMC11973914; doi:10.1021/acs.jpclett.4c03621)
Supplement: Supplementary file 1 — jz4c03621_si_001.pdf [file jz4c03621_si_001.pdf]

Supporting Information: Ultrafast Dynamics and Rearrangement of the EUV Photoacid Generator Phenyl Triflate

Sung Kwon,[†] Jacob Stamm,[†] and Marcos Dantus*,[†,‡,¶]

†*Department of Chemistry, Michigan State University, 48824 East Lansing, MI, United States*

‡*Department of Physics and Astronomy, Michigan State University, 48824 East Lansing, MI, United States*

¶*Department of Electric and Computer Engineering, Michigan State University, 48824 East Lansing, MI, United States*

E-mail: dantus@chemistry.msu.edu

Table of Contents

1 Ion Yield Dynamics **S-2**

2 Individual Residuals **S-3**

3 Timescale Information **S-4**

4 Phase Analysis **S-4**

5 Neutral State Geometry **S-5**

6 Probe Resonance Across Vibrational Modes **S-6**

1 Ion Yield Dynamics

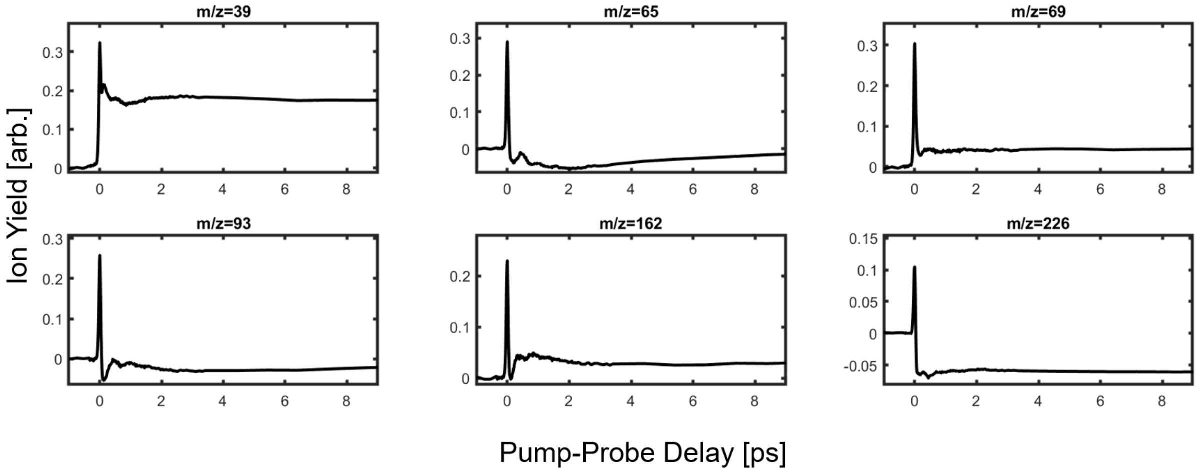

Figure S1: The ion yield as a function of pump-probe delay for the major fragments corresponding to m/z 39, 65, 69, 93, 162, and 226.

2 Individual Residuals

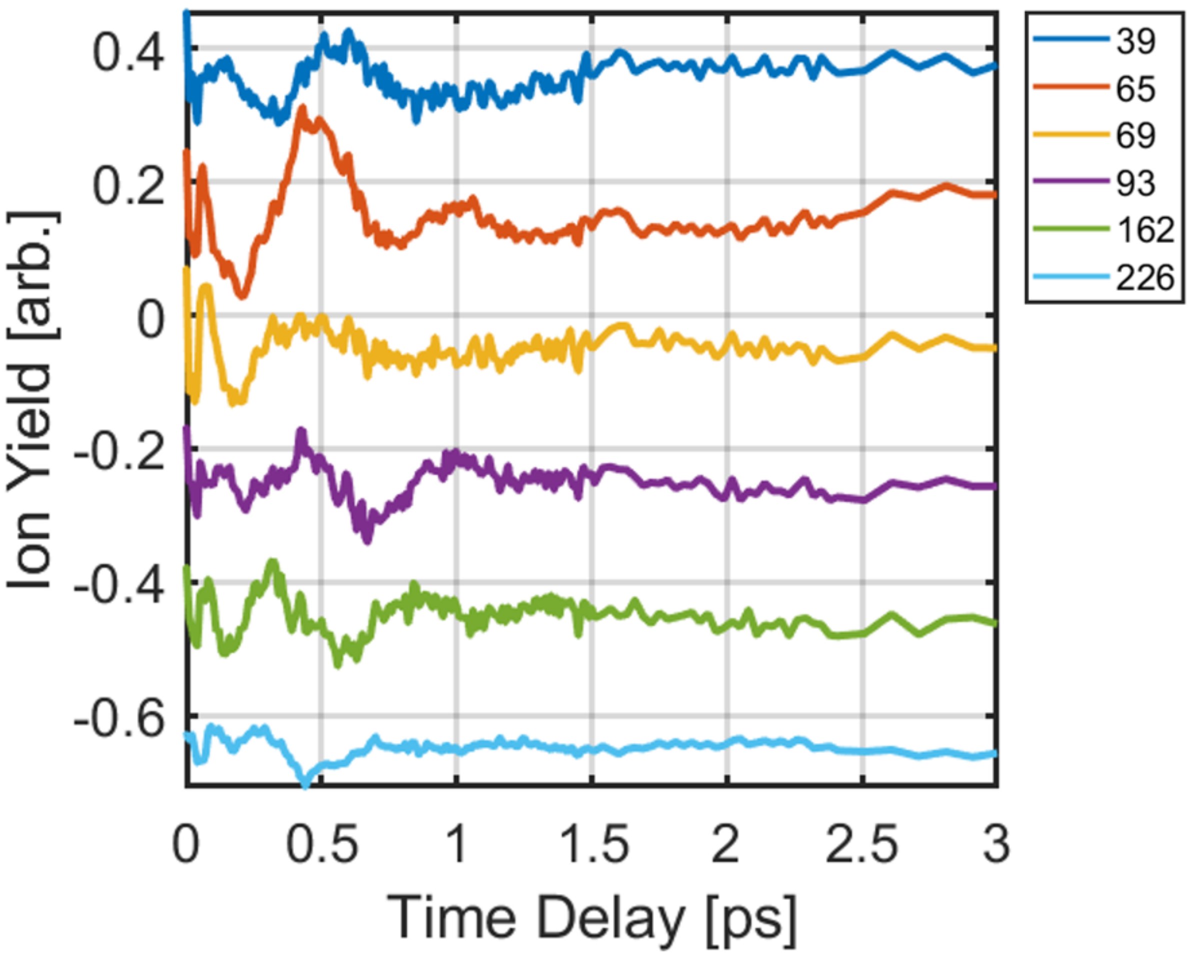

Figure S2: The individual residuals corresponding to m/z 39, 65, 69, 93, 162, and 226. Each residual has been scaled by a factor of eight for clarity.

3 Timescale Information

Table S1: The fit parameters for m/z 226, 162, and 65.

m/z	τ_1 (fs)	τ_2 (fs)	τ_3 (fs)	a_1	a_2	a_3
226	935 ± 70	2940 ± 150	1250 ± 80	0.070	0.076	-0.12
162	160 ± 20	3400 ± 600	4500 ± 1500	-0.08	0.15	-0.14
65	30 ± 5	1460 ± 50	3310 ± 150	0.020	-0.029	-0.087

4 Phase Analysis

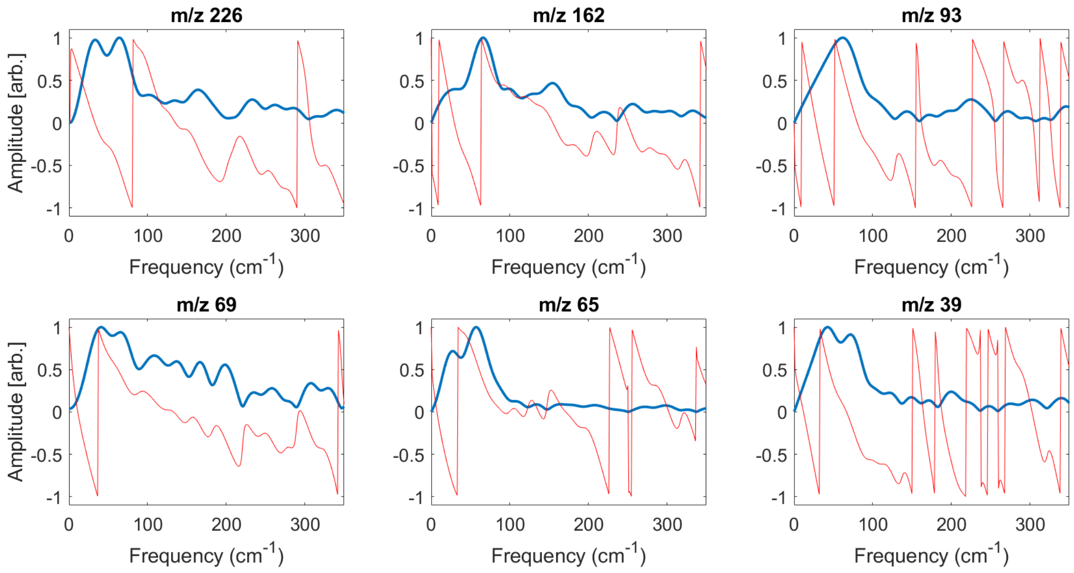

Figure S3: The FFT (blue) and phase (red) of major fragment residuals from the dissociative ionization of phenyl triflate.

5 Neutral State Geometry

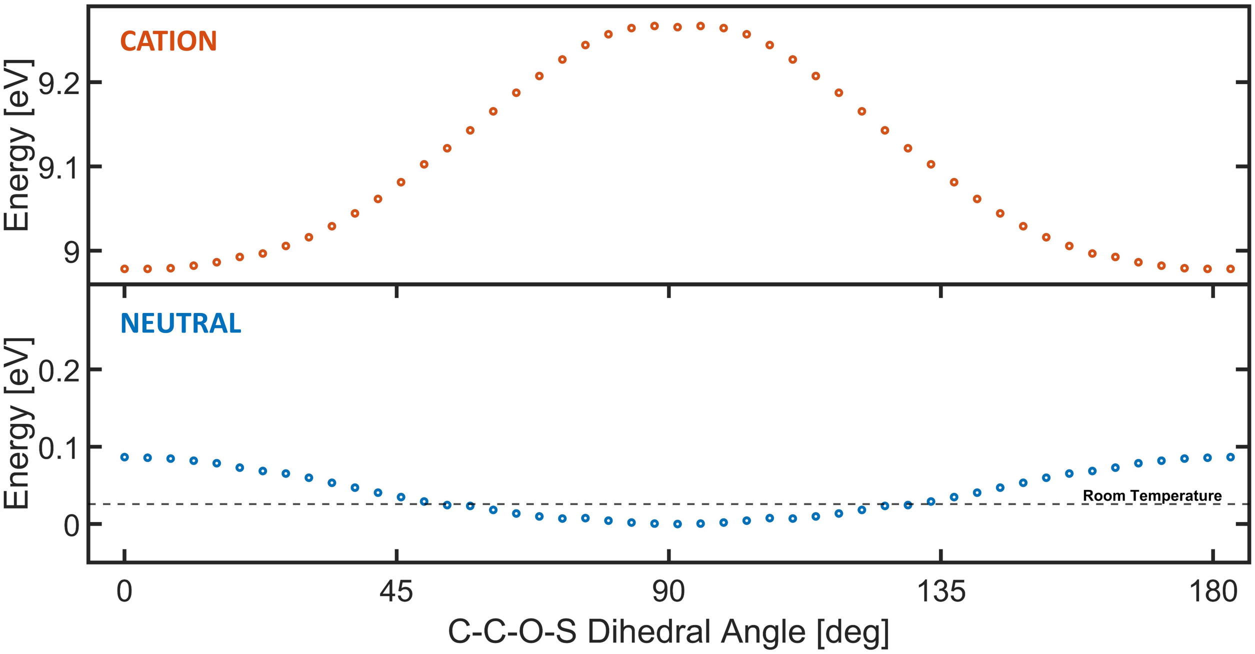

Figure S4: Energy of the neutral and cationic phenyl triflate, relative to its neutral ground state geometry, as a function of phenyl rotation. All other coordinates were allowed to relax across the scan. The kT energy at room temperature is shown as a black line. The calculations were performed at the ωB97X-D/6-311+G** level of theory.

6 Probe Resonance Across Vibrational Modes

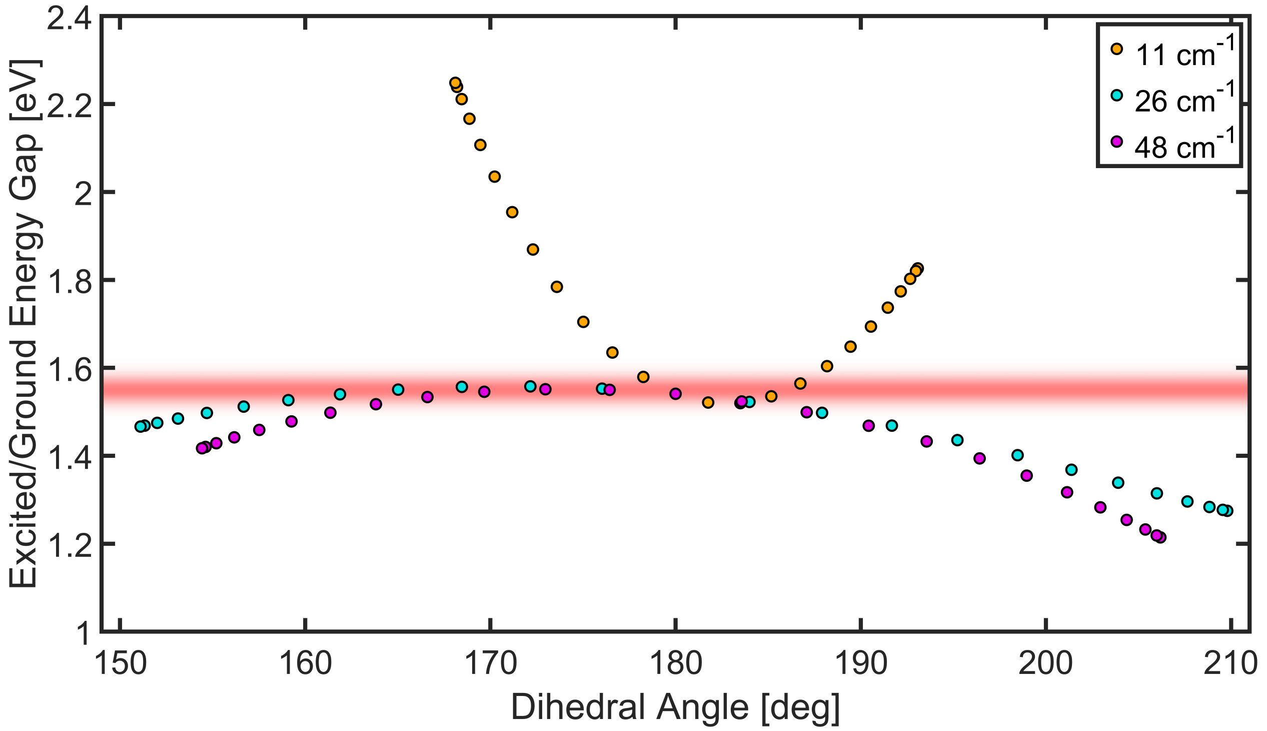

Figure S5: Difference in energy between the ground state and first excited state of the PTF cation as its geometry is changed according to the three lowest vibrational modes. The geometries were identified at the ωB97X-D/6-311+G** level of theory and energies calculated at the M06-2X/cc-PVTZ level of theory. The bandwidth of the laser used in the experiment is shown as a red band.
