# Supplementary material for: Ultrafast Dynamics and Rearrangement of the EUV Photoacid Generator Phenyl Triflate
Source: J Phys Chem Lett. 2025 Mar 27;16(13):3397–401. doi: 10.1021/acs.jpclett.4c03621 (PMC11973914; doi:10.1021/acs.jpclett.4c03621)
Supplement: Supplementary file 2 — jz4c03621_si_002.pdf [file jz4c03621_si_002.pdf]

jz-2024-03621d.R1

Name: Peer Review Information for "Ultrafast Dynamics and Rearrangement of the EUV Photoacid Generator Phenyl Triflate"

First Round of Reviewer Comments

Reviewer: 1

Comments to the Author

This manuscript reports the use of femtosecond time-resolved mass spectrometry to investigate the ultrafast dissociative ionization dynamics of phenyl triflate, a photoacid generator that is used in extreme ultraviolet lithography. Following strong-field ionization by an intense 800-nm laser pulse, the ion yields of various fragments derived from the phenyl triflate cation are recorded as a function of time delay between the ionizing pump pulse and a weak-field, 800-nm probe pulse. The authors detect vibrational wave packet dynamics, with experimental frequencies of ~33 and 66 cm^{-1}, assigned to torsional modes between the phenyl moiety and the triflate group. The observed vibrational wave packet motion is consistent with ab initio calculation results. This manuscript is well-written and I recommend publication of a suitably revised version in The Journal of Physical Chemistry Letters. In their revision, the authors are requested to address the following.

1. The authors have primarily concerned themselves with the analysis of the vibrational wave packet dynamics. The recorded ion yields in Figs. 2a – 2c, however, should also report on the population dynamics. Indeed, these ion yields are fit to a sum of exponential functions. What are the time constants obtained from the fits, and what do these time constants tell us about the dissociation dynamics?

2. On p. 6 lines 5 – 10, the authors state that the “identical phase observed in the yield of these products is consistent with this mechanism because this observation indicates a common transition state barrier acting as a bottleneck for the subsequent fragmentation.” It is not straightforward to decipher the oscillation phases from Fig. S2 because two frequencies contribute to the observed signal. In fact, it appears that the oscillations observed for m/z values of 39 and 162 are slightly phase-shifted relative to the oscillations for m/z values of 65, 69, and 93. It would be clearer if the authors could provide the oscillation frequencies and their corresponding phases for each of the ions shown in Fig. S2.

3. Related to the same sentence in the manuscript that is quoted in the preceding comment, do the authors have any evidence from their ab initio calculations that indicate that the given transition state connects to other fragmentation products?

4. On p. 3 line 58, the authors mention the “coherence map method”, citing ref. 13. For the benefit of the readers, it would be useful if the authors could briefly summarize the key features of the coherence map method.

Reviewer: 2

Comments to the Author

Ultrafast Dynamics and Rearrangement of the EUV Photoacid Generator Phenyl Triflate
Kwon et al. 2025

The manuscript of Kwon et al. addresses the dynamics of strong-field dissociative ionization of phenyl triflate with femtosecond time resolution. It is found that molecular rearrangement and loss of SO_2 occurs within ~2.5 ps. Coherent dynamics are observed in the photoion signal of several fragment ions indicating a common origin. Computations employing density functional theory relate the frequency of the observed modulations to vibrational modes in the cation. Depending on the geometry of the cation at a certain point in time during such a low wavenumber phenyl twisting motion, the probe pulse is either in or out of resonance with the first excited state, which controls whether the transition state for SO_2 loss can be surpassed or not.

1. What is the major advance reported in the paper?

The paper gives insights into the dynamics of the dissociative ionization of a prototypical photoacid generator used in chemically-amplified photoresists for EUV lithography revealing ultrafast dynamics on a picosecond time scale.

2. What is the immediate significance of this advance?

The manuscript shows that ultrafast ionization dynamics are trackable in photoacid generators using strong-field ionization, which should be similar to one-photon processes using EUV light. Insights into these dynamics contribute to a better understanding of the fundamental light-matter interaction in EUV lithography and can potentially contribute to the improvement of photoresist materials.

3. Technical suggestions

p. 2, Introduction

"The shift to shorter wavelengths in microchip technologies has driven the development of new photoacid generators (PAGs),..."

The photoacid generators are actually (almost) identical to what has been used in deep UV lithography. The application and reaction mechanism in EUV lithography is different, though.

It should also be mentioned in the introduction that PAGs are used in chemicallyamplified resists, which have certain advantages in lithography with shorter wavelengths.

p. 2, Introduction

"These regions are then removed through washing,..."

This is only true for positive tone photoresists. In negative tone photoresist, the unexposed regions are removed. PAGs can be used in both types.

p. 2, Introduction

"Studying photoresists in their native condensed phase is challenging,[2]..."

While ref. 2 has some condensed phase data, it mainly focuses on solution phase experiments. More references to the challenges in the condensed phase should be included.

Figure 1

More tightly spaced x-ticks and m/z assignments to the peaks beyond 162 and 226 would help the reader.

p. 3, Results and Discussion

"The cyclopentadienyl cation, Fig. 2c, as well as all other major fragments, shows coherent dynamics that appear to be out of phase with those of the molecular ion,..." Why are the modulations in the ion yield of 162 and 65 also not perfectly in phase? Is it different contribution of the modes to one or the other final ion? If so, how do the phases extracted for a certain frequency from the FT/MEM spectra compare for different fragment ions?

Why is the higher frequency mode more prevalent in m/z=65 and 162 if all have the same transition state?

p. 5, Methods typo in “Multiphoton Intrapulse Inte**r**ference Phase Scan (MIIPS)”

general:
Since the paper is motivated by EUV lithography, the authors might comment on if and how the dynamics might be different by single-photon ionization with EUV light compared to strong-field ionization.

The authors should address the points listed in the technical suggestions, especially the one on the slight dephasing observed for several fragment ions in the ion yields, which is also well visible in the Individual Residuals shown in Figure S2.

Overall, the manuscript is very interesting for the community and well written. After revision, I would recommend it for publication in The Journal of Physical Chemistry Letters.

Author's Response to Peer Review Comments:

RESPONSES TO COMMENTS BY THE REVIEWERS

Comment by Reviewer #1:

The authors have primarily concerned themselves with the analysis of the vibrational wave packet dynamics. The recorded ion yields in Figs. 2a – 2c, however, should also report on the population dynamics. Indeed, these ion yields are fit to a sum of exponential functions. What are the time constants obtained from the fits, and what do these time constants tell us about the dissociation dynamics?

Our response:

We thank the reviewer for pointing out our omission. The fit time constants for the key fragments of PTF are as now summarized in Table SI in the SI.

m/z	τ_1 (fs)	τ_2 (fs)	τ_3 (fs)	a_1	a_2	a_3
226	935 ± 70	2940 ± 150	1250 ± 80	0.070	0.076	-0.12
162	160 ± 20	3400 ± 600	4500 ± 1500	-0.08	0.15	-0.14
65	30 ± 5	1460 ± 50	3310 ± 150	0.020	-0.029	-0.087

The time constants of the fits shown in Fig. 2 are summarized in Supplementary Information Table SI. The fastest dynamics correspond to a 160 fs rise in m/z 162, that we hypothesize is related to the phenyl group rotation into the O-S-C plane. The remaining time constants relate to the 0.5-5 ps dissociation dynamics of this large molecule. A more detailed description of the dynamics will require higher level calculations, and ab initio molecular dynamics simulations. We consider this molecule to be an ideal candidate to be examined by ultrafast X-ray or electron diffraction methods, which are the only experimental tools capable of capturing the motion of the entire molecular structure. Efforts along these lines are already being planned.

Comment by Reviewer #1:

On p. 6 lines 5 – 10, the authors state that the "identical phase observed in the yield of these products is consistent with this mechanism because this observation indicates a common transition state barrier acting as a bottleneck for the subsequent fragmentation." It is not straightforward to decipher the oscillation phases from Fig. S2 because two frequencies contribute to the observed signal. In fact, it appears that the oscillations observed for m/z values of 39 and 162 are slightly phase-shifted relative to the oscillations for m/z values of 65, 69, and 93. It would be clearer if the authors could provide the oscillation frequencies and their corresponding phases for each of the ions shown in Fig. S2.

Our response:

We have extracted the phase for each of the frequencies using fast-Fourier transform analysis. The frequencies and phases are now reported on Table I, and a figure containing the FFT and phase analysis has been added to the SI.

m/z	Frequency 1 (cm^{-1}) ±4	Phase 1 (π) ±0.1 (33 cm^{-1})	Frequency 2 (cm^{-1}) ±4	Phase 2 (π) ±0.1 (64 cm^{-1})
226	33	2.0	64	1.5
162	25	1.9	64	1.0
93	-	-	63	0.5
69	41	1.0	65	0.4
65	28	1.0	56	0.4
39	41	1.0	72	0.0

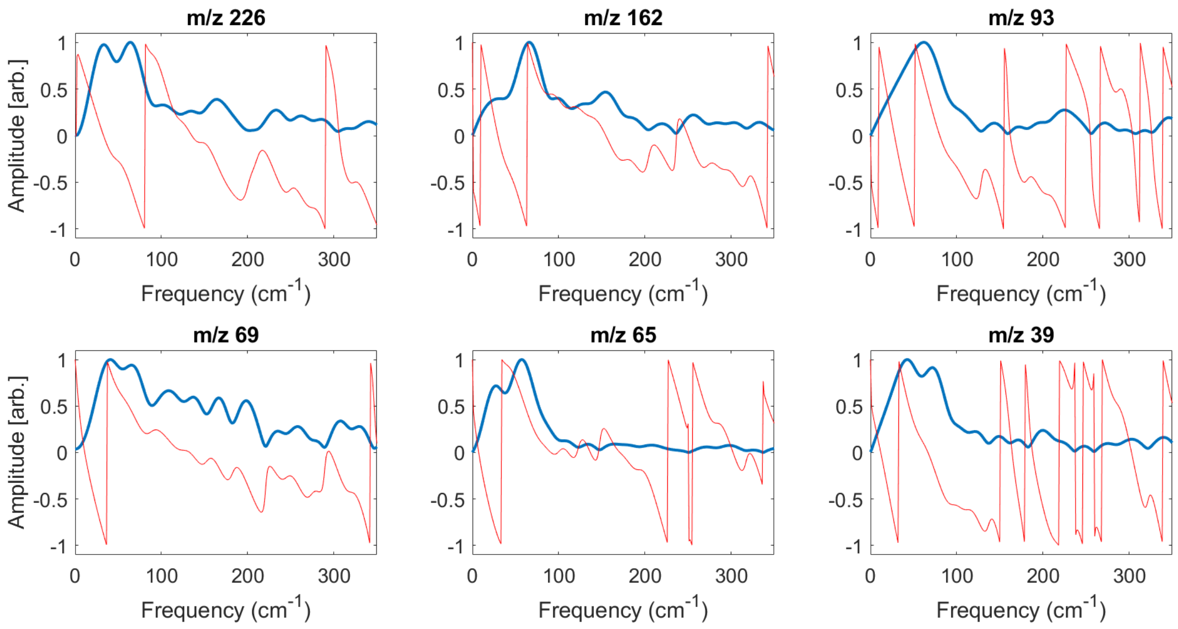

SI Fig. 3. The FFT (blue) and phase (red) of major fragment residuals from the dissociative ionization of phenyl triflate.

Comment by Reviewer #1:

Related to the same sentence in the manuscript that is quoted in the preceding comment, do the authors have any evidence from their ab initio calculations that indicate that the given transition state connects to other fragmentation products?

Our response:

The evidence supporting the transition state identified for SO_2 loss, which links the remaining fragmentation products, comes from the lack of ionization ion and experimental observation of the main frequencies in the pump-probe transients across different product ions (see Table I). From an ab initio perspective, this idea is further supported by the energy release from SO_2 loss (2.3 eV), combined with the reaction progression detailed in Ref. [7] of the main manuscript. The calculations in [7] outline the energetics associated with a series of bond cleavages following SO_2 loss, which leads to the formation of the main product ions discussed in our manuscript.

We have reworded the sentence as follows:

When the phenyl ring is twisted, causing the probe to be out of resonance, excitation becomes less likely, and the molecule does not have enough energy to eliminate SO_2, so it preferentially remains as a molecular ion. However, when the phenyl ring is in the plane of the O-S-C atoms, the fragmentation pathway to eliminate SO_2 can proceed, as the barrier is overcome by the probe energy. We observe a delay or phase shift in the oscillations of different fragment ions, roughly proportional to their size, which we hypothesize is related to the time required to relax from higher excited states reached by the pump to the ground state. Production of the smaller fragment ions requires overcoming barriers of increasing height,[6] and thus requires relaxation from higher excited states with different relaxation times.

Comment by Reviewer #1:

On p. 3 line 58, the authors mention the "coherence map method", citing ref. 13. For the benefit of the readers, it would be useful if the authors could briefly summarize the key features of the coherence map method.

Our response:

We agree that further explanation would benefit the reader. The sentence has been modified in the following way:

The coherence mapping method,[18] which compares the observed vibrational frequencies across product ions to elucidate the intermediate(s) they originate from, was used to analyze the main products.

Comment by Reviewer #2:

p. 2, Introduction

"The shift to shorter wavelengths in microchip technologies has driven the development of new photoacid generators (PAGs),…"

The photoacid generators are actually (almost) identical to what has been used in deep UV lithography. The application and reaction mechanism in EUV lithography is different, though. It should also be mentioned in the introduction that PAGs are used in chemically amplified resists, which have certain advantages in lithography with shorter wavelengths.

Our response:

We have reworded our explanation to more accurately explain the process:

As EUV interacts with the photoresist, it releases photoelectrons that further ionize the film or underlayer.[1-4] The shift to shorter wavelengths in microchip technologies has garnered interest in the chemistry of photoacid generators (PAGs), such as phenyl triflate (PTF), for use in chemically amplified photoresists at these wavelengths. Upon ionization by the secondary electrons, PAGs release acidic groups that promote deprotection and cross-linking, altering the solubility of the exposed regions.

Comment by Reviewer #2:

p. 2, Introduction

"These regions are then removed through washing,…"

This is only true for positive tone photoresists. In negative tone photoresist, the unexposed regions are removed. PAGs can be used in both types.

Our response:

We have corrected the sentence in the following way:

These regions are then either washed away (in positive tone photoresists) or left intact while the unexposed regions are washed away (in negative tone photoresists), creating patterns that can be further processed through doping, etching, or metal filling to construct the chip's intricate three-dimensional structure.

Comment by Reviewer #2:

p. 2, Introduction

"Studying photoresists in their native condensed phase is challenging,2…"

While ref. 2 has some condensed phase data, it mainly focuses on solution phase experiments. More references to the challenges in the condensed phase should be included.

Our response:

We have added a couple of references for the challenges in the condensed phase:

Studying photoresists in their native condensed phase is challenging,[4-6]…"

Laffert, V.; et al. Dissociative photoionization of phenyl triflate, a photoacid generator for photolithography, at 92 eV. The Journal of Chemical Physics 2024, 160, 134303.
Roncaglia, A. Advanced Lithography. *Springer Handbook of Semiconductor Devices*, Springer, 2022, 279–308.

Comment by Reviewer #2:

Figure 1

More tightly spaced x-ticks and m/z assignments to the peaks beyond 162 and 226 would help the reader.

Our response:

Figure 1 has been updated as per the reviewer's suggestion:

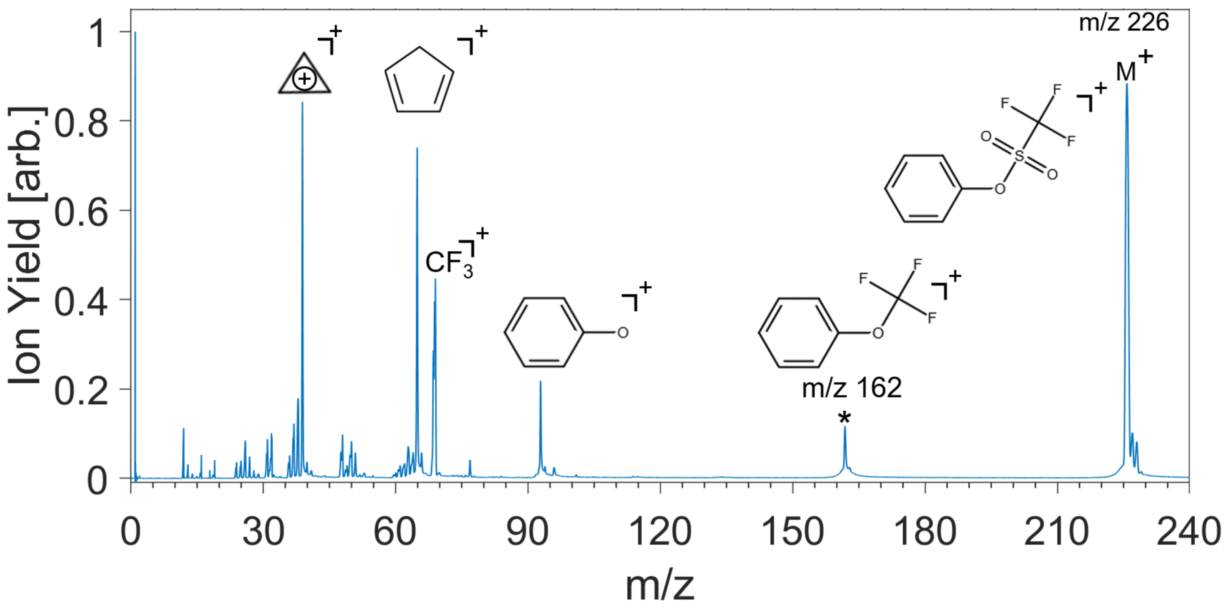

Comment by Reviewer #2:

p. 3, Results and Discussion

"The cyclopentadienyl cation, Fig. 2c, as well as all other major fragments, shows coherent dynamics that appear to be out of phase with those of the molecular ion,…"

Why are the modulations in the ion yield of 162 and 65 also not perfectly in phase? Is it different contribution of the modes to one or the other final ion? If so, how do the phases extracted for a certain frequency from the FT/MEM spectra compare for different fragment ions? Why is the higher frequency mode more prevalent in m/z=65 and 162 if all have the same transition state?

Our response:

m/z	Frequency 1 (cm^{-1}) ±4	Phase 1 (π) ±0.1 (33 cm^{-1})	Frequency 2 (cm^{-1}) ±4	Phase 2 (π) ±0.1 (64 cm^{-1})
226	33	2.0	64	1.5
162	25	1.9	64	1.0
93	-	-	63	0.5
69	41	1.0	65	0.4
65	28	1.0	56	0.4
39	41	1.0	72	0.0

We extracted the phase of the different frequencies using fast Fourier transform analysis (now included in Table I) and added a figure with the analysis to the SI. The main frequency observed in our transients is approximately 64 cm^{-1}. We observe a delay or phase shift in the oscillations of different fragment ions, roughly proportional to their size. Production of the smaller fragment ions requires overcoming barriers of increasing height,[6] and thus requires relaxation from higher excited states with different relaxation times.

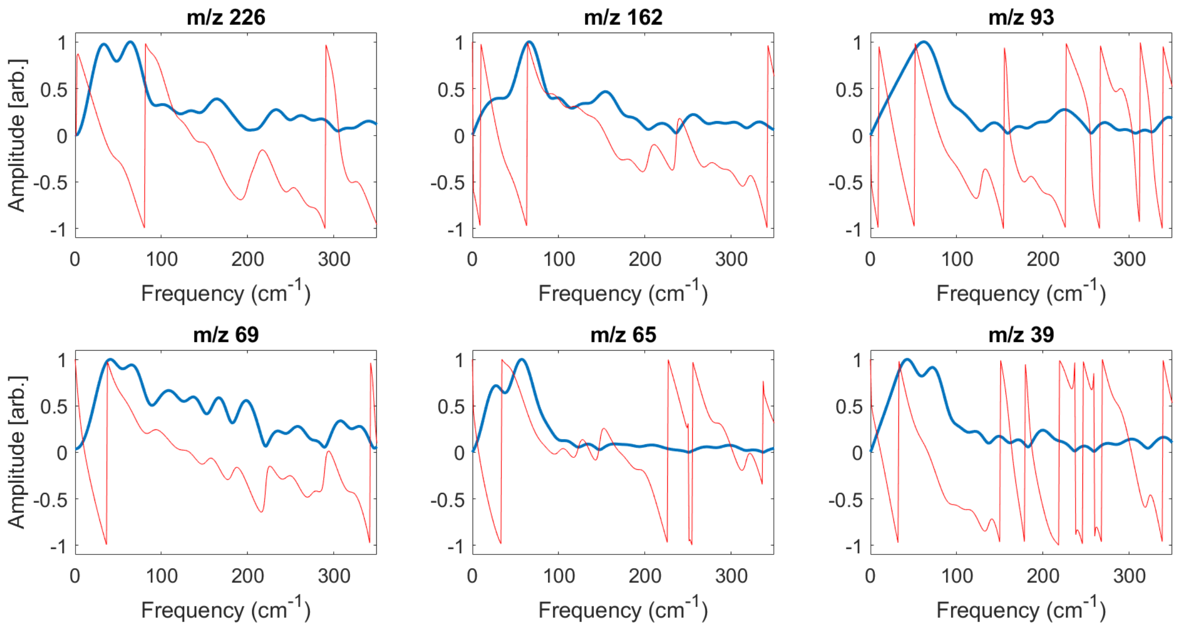

Phase analysis was also carried out (see Fig. S3) and results are summarized in Table 1.

Comment by Reviewer #2:

p. 5, Methods

typo in “Multiphoton Intrapulse Interference Phase Scan (MIIPS)”

Our response:

The typo has been corrected:

Multiphoton Intrapulse Interference Phase Scan (MIIPS)

Comment by Reviewer #2:

general:

Since the paper is motivated by EUV lithography, the authors might comment on if and how the dynamics might be different by single-photon ionization with EUV light compared to strong-field ionization.

Our response:

We agree that an explanation of how our measurements are relevant to EUV photolithography is warranted. In EUV photolithography, secondary electrons released by the ionizing light are responsible for the chemical transformations.[1-3] While the ionization cross section for electrons peaks at 70 eV, secondary electrons span a broad range of energies. Therefore, an ionization process such as strong-field ionization that produces ions with a wide range of internal energies most effectively simulates the chemical activation that occurs in EUV photolithography.

The following sentence has been added to the third paragraph of the introduction:

Our group has been exploring the ultrafast chemical processes that occur following the interaction of molecules with secondary electrons. Here, we examine the ultrafast dissociative dynamics of ionized PTF with femtosecond time resolution. Strong-field ionization, which generates ions with a wide range of internal energies, effectively simulates the chemical activation seen in EUV photolithography, where secondary electrons with a broad range of energies play a key role.[1-3] Our findings offer new insights into the mechanisms at play within photoresists under high-energy conditions, with a particular emphasis on the ultrafast chemistry occurring immediately after ionization, before collisions with nearby molecules become significant.

The following text has been added after the introduction of the mass spectrum in the Results and Discussion section:

… further supports the prevalence of the intramolecular rearrangement. Differences between the spectra obtained here and by 92 eV single-photon excitation [6], such as a greater abundance of the molecular ion as well as a greater abundance of cyclopropenium, arise from the broad range of intramolecular energies resulting from strong-field ionization. Recall that in EUV photolithography the majority of chemical transformations are induced by secondary electrons and that the electron cross section peaks at 70 eV. To mimic 70 eV electron-ionization mass spectra, it has been found that one needs 20 eV photons.[15] Here we propose that strong-field ionization, is best for mimicking the broad range of ionization energies occurring in EUV photolithography. The use of femtosecond near IR pulses, satisfies this requirement and when coupled with disruptive probing [16] can provide valuable information regarding the fragmentation dynamics of photoacid generators such as PTF.

jz-2024-03621d.R2

Name: Peer Review Information for "Ultrafast Dynamics and Rearrangement of the EUV Photoacid Generator Phenyl Triflate"

Second Round of Reviewer Comments

Reviewer: 1

Comments to the Author

The authors have addressed the reviewers' comments in a satisfactory manner. I recommend publication of the manuscript in its current form.

Reviewer: 2

Comments to the Author

The authors have addressed all my comments and the additions to the manuscript have led to a significant improvement.

I therefore suggest to accept this manuscript for publication in The Journal of Physical Chemistry Letters.

Author's Response to Peer Review Comments:

Dear Editor,

Thank you for your 12-Mar-2025 email indicating that the manuscript is ready except for a brief description of the supplementary material.

We have added this description before the references.

Best regards,

The authors.
